# Air Temperature Error Correction Based on Solar Radiation in an Economical Meteorological Wireless Sensor Network

**DOI:** 10.3390/s150818114

**Published:** 2015-07-24

**Authors:** Xingming Sun, Shuangshuang Yan, Baowei Wang, Li Xia, Qi Liu, Hui Zhang

**Affiliations:** 1 Jiangsu Engineering Center of Network Monitoring, Nanjing University of Information Science & Technology, Nanjing 210044, China; E-Mails: sunnudt@163.com (X.S.); qrankl@163.com (Q.L.); 2 College of Computer & Software, Nanjing University of Information Science & Technology, Nanjing 210044, China; 3 Jiangsu Collaborative Innovation Center on Atmospheric Environment and Equipment Technology, Nanjing 210044, China; 4 Collaborative Innovation Center on Forecast and Evaluation of Meteorological Disasters, Nanjing University of Information Science & Technology, Nanjing 210044, China; E-Mail: xialixf@163.com; 5 Huaiyin Institute of Technology, Huaian 223003, China; E-Mail: baobaomami@sina.com

**Keywords:** air temperature, solar radiation, wireless sensor networks, data correction

## Abstract

Air temperature (AT) is an extremely vital factor in meteorology, agriculture, military, *etc.*, being used for the prediction of weather disasters, such as drought, flood, frost, *etc.* Many efforts have been made to monitor the temperature of the atmosphere, like automatic weather stations (AWS). Nevertheless, due to the high cost of specialized AT sensors, they cannot be deployed within a large spatial density. A novel method named the meteorology wireless sensor network relying on a sensing node has been proposed for the purpose of reducing the cost of AT monitoring. However, the temperature sensor on the sensing node can be easily influenced by environmental factors. Previous research has confirmed that there is a close relation between AT and solar radiation (SR). Therefore, this paper presents a method to decrease the error of sensed AT, taking SR into consideration. In this work, we analyzed all of the collected data of AT and SR in May 2014 and found the numerical correspondence between AT error (ATE) and SR. This corresponding relation was used to calculate real-time ATE according to real-time SR and to correct the error of AT in other months.

## 1. Introduction

Meteorology monitoring is highly required in many domains, e.g., weather forecasting, agricultural, traffic [1], *etc.* Unexpected meteorological calamities, such as floods, bring much damage to our community and ourselves. Thus, it is imperative for us to take drastic measures by monitoring meteorological factors and forecasting weather disasters.

The prosperity of sensors and automated devices has fostered the feasibility of automatic meteorological observation [2] applications, in which air temperature (AT) [3,4] is regarded as one of the most significant meteorological factors [5].

In recent years, numerous approaches have been presented for AT monitoring, in which a conventional scheme, called automatic weather stations (AWS) [6,7,8], has been applied widely in the meteorological departments all over the world. Based on the use of meteorological temperature sensors, accurate AT data can be collected. However, a meteorological sensor costs too much to be applied in a large-scale scenario. HMP45D is a typical AT sensor used in AWS with its price ranging from 500 to 600 dollars. Plus, an AWS will cost more to guarantee the accuracy of AT sensing due to the requirement of additional facilities, such as a thermometer screen. It accordingly becomes challenging when applying a high-density AWS system for weather disaster monitoring.

In order to reduce the cost and to advance the flexibility of AT observation, wireless sensor networks (WSNs) [9,10,11,12,13] have been take into consideration. During the last decade, WSNs have been broadly utilized in various fields, including air temperature observation [8], water monitoring [14,15,16,17], forest monitoring [18,19], industrial monitoring [20,21], agriculture monitoring [22,23], battlefield surveillance [24,25], intelligent transportation [26,27], smart homes [28], animal behavior monitoring [29,30] and disaster prevention [31,32]. The high flexibility of WSNs makes it possible to deploy nodes swiftly in a disaster spot, and the low cost of the sensing node provides the possibility to control the cost of AT monitoring at an acceptable level.

Due to these advantages of WSNs, we established a meteorological WSN using our own sensing nodes to collect data of AT and other meteorological factors in a practical environment, involving our campus and other weather stations of meteorological departments. These exquisite sensing nodes can be deployed in a variety of locations without additional infrastructure. In addition, as a low-cost temperature sensor, SHT15 [33] is embedded in our sensing node for both air relative humidity and temperature sensing, which successfully reduces the cost of the specialized temperature sensor from over $500 to $5.

At the same time, however, the accuracy of SHT15 is influenced by numerous external factors, such as solar radiation (SR) [34,35,36], precipitation [3,37], wind [37], *etc.* Deviations [38] happen between AT values collected by the sensing node (NodeAT) and actual ones, which require correction.

Early work in [1] has given an approach to improve the accuracy of AT collected by SHT15 in a WSN. The method depends on the principle of a back propagation (BP) neural network [1,5,39]. Having improved data accuracy, this method suffers from the establishment of a data correction model by training data for days under similar weather conditions [40], which cannot fit all seasons or weather conditions. Even in the same month, it has to build different data correction models corresponding to the weather conditions [1]. Real-time data correction becomes a challenge via that approach.

Previous studies have shown that AT is determined by many factors [3,41]. Solar radiation, surface net radiation, advection and heat storage are related to the variation of AT, but SR drives other components as a forcing factor and is found to be closely correlated with AT, which therefore is considered to be the key factor in our correcting method of AT.

There are various radiometric quantities [34,42,43] to represent SR (refer to Appendix A.1), but we only adopt irradiation [44,45] as the measurement of SR in this work. Irradiation refers to the quantity of solar energy arriving at a surface during a given period of time, and the unit of irradiation is kJ·m${}^{-2}$·h${}^{-1}$ or MJ·m${}^{-2}$·day${}^{-1}$. Radiation is employed in a generic sense, and it stands for irradiation in this paper.

Furthermore, There are two kinds of radiation that reach the Earth from the Sun through the atmosphere (refer to Appendix A.2). One is direct radiation, and the other is diffuse radiation. Direct radiation [44] is the radiation arriving on the ground directly in line of the solar disk, which is strong under cloudless skies and low under cloudy skies. Diffuse radiation [44] is the solar radiation arriving at the Earth’s surface scattered by air molecules, aerosols and clouds. The quantity of total direct and diffuse radiation reaching the ground is very vital to the temperature variation on the Earth’s surface. The sensing node used in the project suffers not only from direct radiation, but also diffuse radiation due to the outdoor arrangement. Thus, it is necessary to take both direct and diffuse radiation into account in the experiment. Global solar radiation [44] is the sum of the direct plus diffuse radiation on a horizontal surface. It can be used to describe the total radiation and is measured by pyranometers, which are radiometers with hemispherical fields of view. The data of SR collected by AWS (AwsSR) and used in this experiment is hourly global solar irradiation. No matter how the air condition is, global solar radiation is the real radiation, which the pyranometer measures and from which the sensing node suffers.

Besides, the standard data of air temperature and solar radiation used in this study are all collected by the AWS at Nanjing University of Information Science and Technology (NUIST). This AWS was founded according to the AWS construction technical standard and has a national base station Number 59606. The measurements in the AWS also follow the requirements of the World Meteorological Organization (WMO) and can be treated as correctly measured data. What is more, the sensing node with ID Number 105 used in this study is placed in the AWS. Geographically, the location of node No. 105 is close to the pyranometer, which is used to sense solar radiation in the AWS, so we can treat solar radiation collected by the AWS as the same as that from which the node suffered. Hence, AT collected by the AWS (AwsAT) can be treated as corrected AT, and AwsSR can be treated as the standard SR in the experiment.

**Figure 1:**
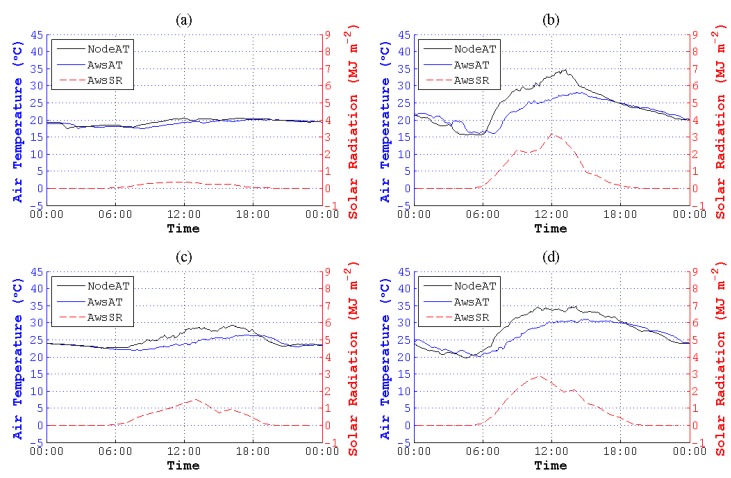
Air temperature (AT) values collected by the sensing node (NodeAT), AT collected by the automatic weather station (AWS) (AwsAT) and AwsSR (SR, solar radiation) sensed directly by different kinds of sensors on four days in May. (**a**) Thundershowers with early/moderate rain late on 10 May 2014; (**b**) cloudy on 13 May 2014; (**c**) showers on 25 May 2014; (**d**) clouds that are early/clearing late on 27 May 2014. The SRs in (**a**,**c**) were weak compared to the SRs in (**b**,**d**), which were in fine weather. Though there are different weather conditions, the trend of NodeAT is similar to AwsSR. Deviations between NodeAT and AwsAT are increasing or decreasing along with the rise or reduction of AwsSR.

According to the figures of NodeAT, AwsAT and AwsSR on the 10th, 13th, 25th and 27th day with different weather conditions in May, NodeAT is closely related to AwsSR, as is shown in Figure 1.

It is quite reasonable to take SR into account in the research of NodeAT correction. In this work, we found the relevance among NodeAT, AwsAT and AwsSR and proposed an original approach to reduce the error of NodeAT based on the value of SR.

This work is motivated by our real-time [11] meteorological factor collecting project at NUIST. We launched an ongoing WSN consisting of dozens of sensing nodes continuously collecting scientific data. In this application, sensing nodes have to sustain solar radiation, for they are arranged in an open area. Therefore, it is urgent to discover the relation among NodeAT, AwsAT and AwsSR and to invent an effective method to correct the value of NodeAT.

## 2. Methodology

### 2.1. Overview

There are two key parts in our methodology: one is data processing and analyzing, and the other one is data correcting.

In the first part, the data of NodeAT, AwsAT and AwsSR are processed and analyzed to get intermediate data, like NodeATinterp (interpolated NodeAT), NodeATinterpshift (shifted NodeATinterp), *etc.* These intermediate data are used as the medium to achieve the functional relationship of AT error (ATE) and SR and to get the ATE-SR function eventually.

Then, in the second part, the errors of NodeAT are corrected by using the ATE-SR function and AwsSR. There are three key correcting procedures: (1) correcting the time coordinates of NodeAT to eliminate the time phase difference between NodeAT and AwsAT; (2) calculating the theoretical AT error through the ATE-SR function according to real-time AwsSR; and (3) subtracting the AT error acquired in (2) from the AT data obtained in (1) to achieve the corrected AT.

### 2.2. Terms

In order to make it convenient to state the method, several accessible abbreviations of terms were defined in this paper, as is presented in Table 1.

**Table 1:** All terms involved in the manuscript have two forms. One is the generic form, as is listed in the column “Abbreviations”, which is used generally in the text. The other is the mathematical form, as is listed in the column “Mathematical Forms”, which is used to state the course of the methodology. Terms with the suffix *interp* mean that the data are processed by interpolation and with the suffix *shift* mean that the data are processed by time shifting. NodeAT, AwsAT, AwsSR, NodeATinterp, AwsATinterp, AwsSRinterp, NodeATinterpshift, AwsSRinterpshift, NodeATE, NodeATtime, AwsATtime, AwsSRtime and Interptime are involved in the part for the data preprocessing and analysis. NodeAT, AwsSR, NodeATshift, AwsSRshift, CalcATE and NodeATcorr are used in the part for data correction.

Descriptions	Abbreviations	Mathematical Forms
AT collected by Node	NodeAT	*Tnode*
AT collected by AWS	AwsAT	*Taws*
SR collected by AWS	AwsSR	*Raws*
Interpolated NodeAT	NodeATinterp	*TInode*
Interpolated AwsAT	AwsATinterp	*TIaws*
Interpolated AwsSR	AwsSRinterp	*RIaws*
Shifted NodeATinterp	NodeATinterpshift	*TISnode*
Shifted AwsSRinterp	AwsSRinterpshift	*RISaws*
Shifted NodeAT	NodeATshift	*TSnode*
Shifted AwsSR	AwsSRshift	*RSaws*
Deviation between NodeATinterpshift and AwsATinterp	NodeATE	*TEnode*
ATE Calculated by using ATE-SR function	CalcATE	*TEcalc*
Corrected NodeAT	NodeATcorr	*TCnode*
sample points of time corresponding to NodeAT	NodeATtime	*ttnode*
sample points of time corresponding to AwsAT	AwsATtime	*ttaws*
sample points of time corresponding to AwsSR	AwsSRtime	*traws*
sample points of time corresponding to interpolated data	Interptime	*tinterp*

### 2.3. Framework

**Figure 2:**
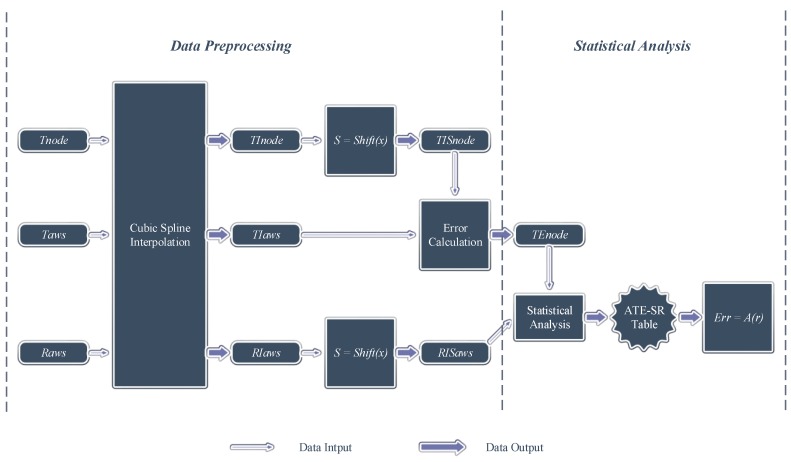
The procedure of data preprocessing and analysis.

**Figure 3:**
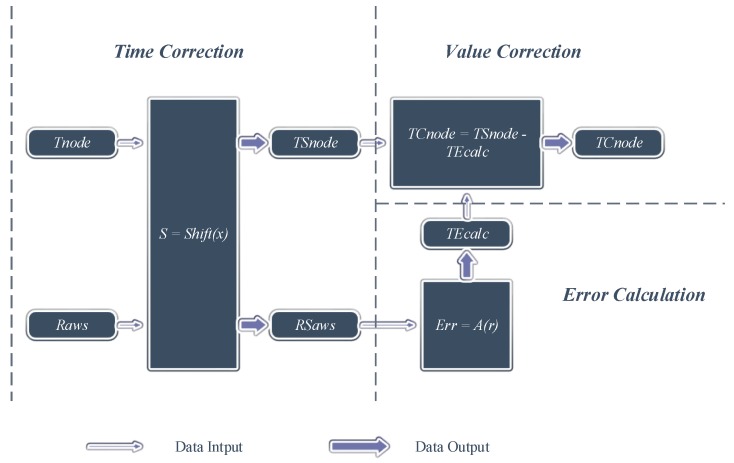
The procedure of data correction.

There are two key steps in the part for the data preprocessing and analysis and three steps in the part for the data correction, as is depicted in Figure 2 and Figure 3.

In Figure 2, cubic spline interpolation is applied to process raw data Tnode, Taws and Raws and to get interpolated data TInode, TIaws and RIaws. Then, the time coordinates of TInode and RIaws are shifted to get TISnode and RISaws. TISnode and TIaws are used to compute TEnode. Lastly, an ATE-SR tabular function Err=A(r) is acquired by the correlation analysis of TEnode and RISaws.

In Figure 3, TSnode is obtained by shifting the time coordinate of Tnode to decrease the time deviation between Tnode and Taws. RSaws is also obtained by shifting the time coordinate of Raws to fit the time coordinate of TSnode. Then, TEcalc is calculated by using RSaws as the parameter of the ATE-SR function Err=A(r). Lastly, TSnode and TEcalc are used to calculate TCnode.

### 2.4. Data Preprocessing

#### 2.4.1. Interpolation

Due to the different sampling frequencies of NodeAT, AwsAT and AwsSR, we use the methods of interpolation to regulate these data at the same time sampling points. It is justified to describe the principle of interpolation used in our work by referring to Appendix B.1. Moreover, the cubic spline function is elected as the interpolation function to process raw data in our experiment. The advantages of the cubic spline function can be seen in Appendix B.2.

Suppose that the function Tnode=f(ttnode) is known at the M+1 points $\left(ttnode_{0},Tnode_{0}\right)$, ..., $\left(ttnode_{M},Tnode_{M}\right)$, where the values $ttnode_{k}$ are spread out over the interval [a,b] and satisfy:

$$a\leq ttnode_{0}<ttnode_{1}<\cdots <ttnode_{M}\leq b\;\text{and}\;Tnode_{k}=f\left(ttnode_{k}\right)$$

Then, a function TInode=F(tinterp) is established at the L+1 points $\left(tinterp_{0},Tnode_{0}\right)$, ..., $\left(tinterp_{L},Tnode_{L}\right)$, where the values $tinterp_{k}$ are spread out over the same interval [a,b] of $ttnode_{k}$, but satisfy:

$$a\leq tinterp_{0}<tinterp_{1}<\cdots <tinterp_{L}\leq b\;\text{and}\;TInode_{k}=F\left(tinterp_{k}\right)$$

F(tinterp) is the piecewise polynomial constructed by the function of cubic spline interpolation and is used to approximate f(ttnode) over the entire interval [a,b].

Similarly, functions TIaws=G(tinterp), RIaws=H(tinterp) can be established at the L+1 points of time $tinterp\in \text{Φ}=\left\{tinterp_{0},tinterp_{1},...,tinterp_{L}\right\}\subseteq \left[a,b\right]$ in order to approximate corresponding functions Taws=g(ttaws), Raws=h(traws). Then, the sampling points of TInode, TIaws and RIaws are unified.

The cubic spline interpolation applied in our practical work is a good method to process row data of AT and SR, which can be fortunately acquired in MATLAB as a built-in function:

vq=interp1(x,v,xq)

In this function, it returns interpolated values of a 1D function at specific query points using spline interpolation. Vector *x* contains the sample points, and *v* contains the corresponding values v(x). Vector xq contains the coordinates of the query points [46]. For example, we can get TInode by using this function:

TInode=interp1(ttnode,Tnode,tinterp)

#### 2.4.2. Time Shift

Due to the outdoor arrangement of nodes, they were affected by radiation, involving solar (or short-wave) radiation [35], terrestrial (or long-wave) radiation [47], atmospheric radiation [47], *etc.* Additionally, the integrated sensor in the sensing node can be influenced by solar radiation by a significant measure. The trend of NodeAT and AwsSR in Figure 1 also shows that NodeAT changes along with AwsSR. However, specialized temperature sensors in the thermometer do not suffer from solar radiation. AwsAT, which is sensed by the specialized sensors, can be regarded as the actual temperature. Moreover, AT is an indicatrix of the energy of the atmosphere, and the atmospheric energy mostly derives from solar radiation. Due to the duration of the transmission of energy from solar radiation to the atmosphere, there is a sensing delay between the low-cost sensor and the specialized sensor. In conclusion, the trend of NodeAT is synchronized with AwsSR, but there is a phase difference between NodeAT and AwsAT. Hence, it is imperative for us to transform the time coordinate of TInode and RIaws to fit TIaws.

Suppose that the function S=Shift(x) is used to transform the time coordinate. Then, there is no doubt of getting the function TISnode=FS(t) by the transformation as follows:

$$\begin{array}{cc} TISnode & =Shift(TInode)\\ & =Shift(F(t))\\ & =FS(t),\;\;\;t\in \text{Φ} \end{array}$$

Similarly,

$$\begin{array}{cc} RISaws & =HS(t),\;\;\;t\in \text{Φ} \end{array}$$

#### 2.4.3. Error Calculating

The data processing executed above makes it possible to calculate the authentic deviation between NodeAT and AwsAT. TEnode can be obtained as follows:

$$\begin{array}{cc} TEnode & =TISnode-TIaws\\ & =FS(t)-G(t)\\ & =E(t),\;\;\;\;t\in \text{Φ} \end{array}$$

### 2.5. Statistical Analysis

Comparing TEnode and RISaws, we can find that there is a strong relevance between them. Therefore, it is conceivable to gain a numerical correspondence between them.

The corresponding relation of ATE and SR is established by using statistical analysis. Theoretically, every value of SR should map a single value of ATE, but there are several ATE values corresponding to one SR value in the real experiment. Thus, it is necessary to do some statistical analysis to obtain a single valued mapping from SR to ATE. We calculated the average of ATE corresponding to every possible value of SR (from 0.01 to 3.60 increasing by 0.01) in May and acquired a tabulation between ATE and SR. Then, the ATE-SR function Err=A(r) at r∈Ψ={0.01,0.01,...,3.60} is constituted based on this tabulation, where *r* is every possible value of SR in May and Err is the homologous ATE. The real-time ATE can be calculated by using real-time SR as the input parameter *r* in Err=A(r).

### 2.6. Correction

#### 2.6.1. Time Correction

The time coordinate of Tnode and Raws can be corrected by using function S=Shift(x), and TSnode and RSaws can be achieved simultaneously. Thus, the trends of TSnode, RSaws and Taws are synchronal to each other.

#### 2.6.2. Error Calculation

The value of TEcalc can be calculated according to the value of RSaws, using the ATE-SR function Err=A(r):

TEcalc=A(RSaws)

#### 2.6.3. Value Correction

It is possible to get the corrected AT TCnode:

$$\begin{array}{cc} TCnode & =TSnode-TEcalc\\ & =TSnode-A(RSaws) \end{array}$$

## 3. Experimental Section

### 3.1. Experiment Foundation

**Figure 4:**
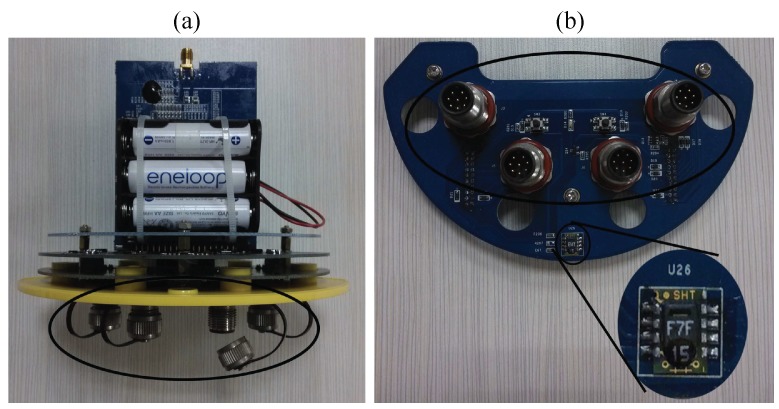
Sensing nodes and the deployment at Nanjing University of Information Science and Technology (NUIST). (**a**) The circuit board inside of the sensing node; (**b**) the bottom circuit board containing the on-board sensor SHT15 and providing interface circuits for external sensors.

We designed the sensing nodes and carried out the ongoing WSN of the meteorological factor monitoring project by using these nodes. Our sensing nodes were designed based on the technology of WSN, on-board sensor, ZigBee, integrated circuit, *etc.* The sensing node is also equipped with a solar panel to guarantee the supply of electric power. The sensing node can generate electricity for its battery pack in the daytime and consume reserved electric energy in the evening. That is also the reason why we tend to arrange the node in open air. We have deployed several meteorological WSNs in Beijing, Xi’an, Wuhan, Changsha and Nanjing in China. We can use the on-board temperature and relative humidity sensor integrated in the node to sense real-time AT. Besides the on-board sensor, the node also can be connected to specialized meteorological sensors, like an AT sensor, anemometer, pyranometer, *etc.*, to collect different kinds of meteorological factors. Figure 4a presents the internal circuit structure of the sensing node, which contains the power module, on-board sensor, interface circuits, *etc.* We adopt SHT15 as the on-board temperature sensor, which is integrated on the bottom circuit board, as is shown in Figure 4b. In Figure 4a,b, the universal interfaces in the node are highlighted by the big ellipses. It is convenient for users to connect external meteorological sensors though these ports. However, for saving on the costs of the project, we adopt the on-board sensor to collect AT in most sensing nodes.

**Figure 5:**
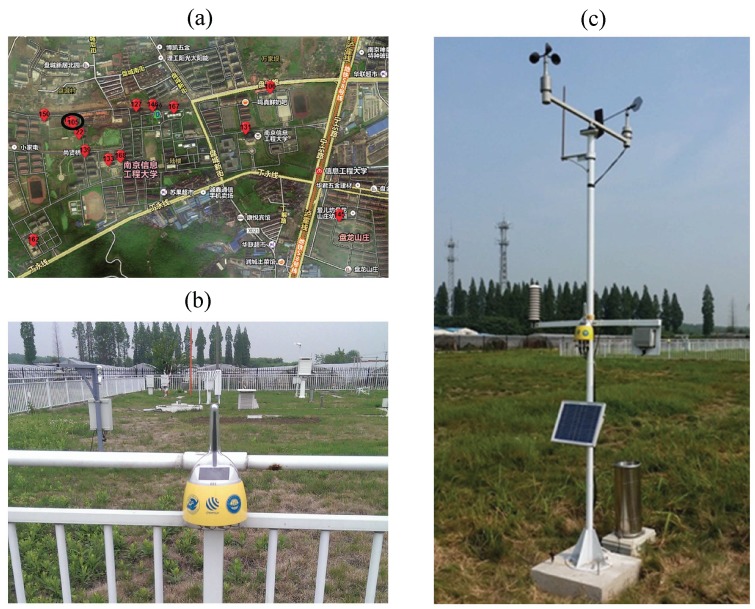
Sensing nodes and the deployment at NUIST. (**a**) The state of the nodes’ deployment; (**b**) a sensing node collecting AT through the on-board sensor; (**c**) a sensing node connected to several meteorological sensors.

NodeAT employed in this experiment was sensed by the on-board temperature sensor in node No. 105. This node was contained in the wireless meteorological sensing network deployed in the campus of NUIST, as is shown in Figure 5a.

The numerical labels marked on the map represent every node deployed in the campus. We circle the label of node No. 105 with the small ellipse in Figure 5a. This node was deployed in the AWS of NUIST. Owing to the SR sensing work being conducted with the AWS, we can apply AwsSR as the same one as node No. 105 suffered. Figure 5b shows a single node that just collects AT by using the on-board temperature sensor. The node in Figure 5c is connected to the anemometer, pluviometer and other meteorological sensors to collect numerous varieties of meteorological factors. This meteorological WSN at NUIST has been set up since August 2013 and has been collecting meteorological data continuously. There are sufficient data for us to execute the experiment.

**Figure 6:**
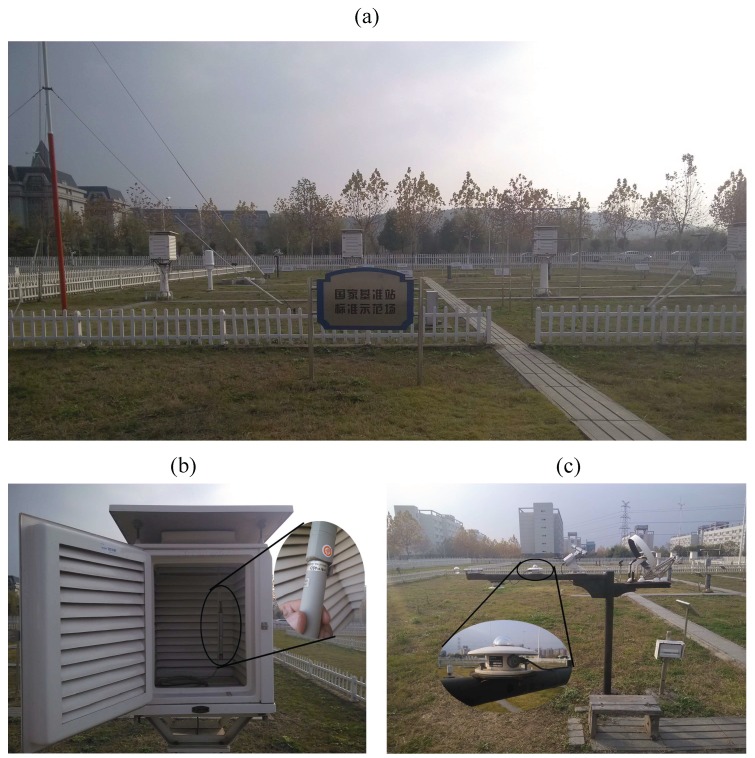
National weather station at NUIST, China, No. 59606. (**a**) Panorama of the AWS; (**b**) the meteorological temperature sensor (HMP45D) in the thermometer screen in the AWS; (**c**) the pyranometer (TBQ-2-B) in the AWS.

This research also relies on the standard data supply of the AWS at NUIST. The AWS at NUIST (depicted in Figure 6a) is a national base station, whose number is 59606. This weather station was founded according to the AWS construction technical standard. Thus, meteorological data applied to it can be treated as the standard data. It is equipped with all the infrastructure demanded by a standard weather station. The meteorological AT sensor used in this AWS is HMP45D (Figure 6b), which is placed in the thermometer screen to avoid solar radiation. The pyranometer TBQ-2-B (Figure 6c) is applied in the AWS to sense global SR.

In the actual experiment, there are three kinds of raw data acting as the basic data: (1) NodeAT sensed by SHT15 in node No. 105; (2) AwsAT sensed by the HMP45D in the AWS; and (3) AwsSR sensed by the TBQ-2-B in the AWS. We first carried out the course of data preprocessing and analysis in May and obtained ATE-SR tabulation, then accomplished the correction of NodeAT in other months and, lastly, did a performance evaluation to evaluate the efficiency of the method. The analysis work was based on the data of NodeAT, AwsAT and AwsSR collected directly by sensors in May. After this procedure, ATE-SR tabulation was established and was used to correct NodeAT sensed by node No. 105 in June to December, employing AwsSR as an input parameter.

### 3.2. Data Process and Correction

All of the meteorological data involved in this research in May had to be dealt with to fulfill the course of data preprocessing and analysis. However, we only took one day (13 May 2014) to demonstrate the course of data processing.

**Figure 7:**
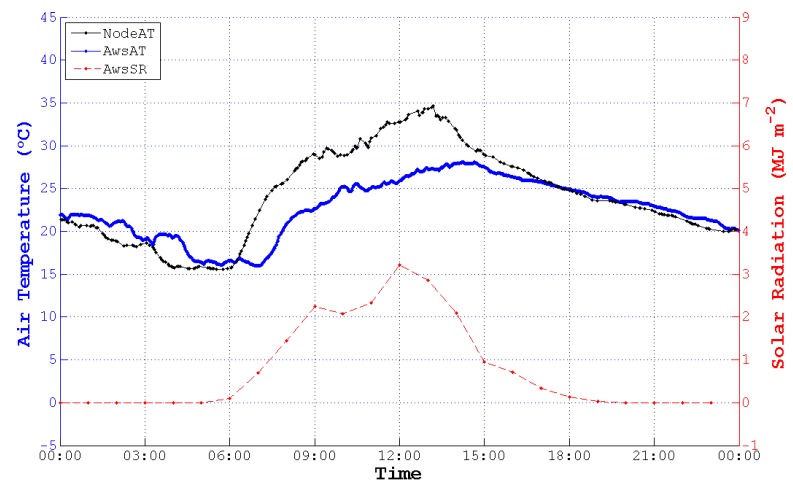
Raw data of NodeAT, AwsAT and AwsSR sensed directly by sensors on 13 May 2014.

As is symbolically illustrated in Figure 7, the sample points of NodeAT, AwsAT and AwsSR are different from each other. It is difficult for us to calculate the deviation between NodeAT and AwsAT. Moreover, the accumulative time interval of AwsSR is 60 min. The sample points of AwsSR are too few to accomplish the analysis. In order to carry out the statistical analysis between ATE and SR, we must acquire more sample points of SR.

**Figure 8:**
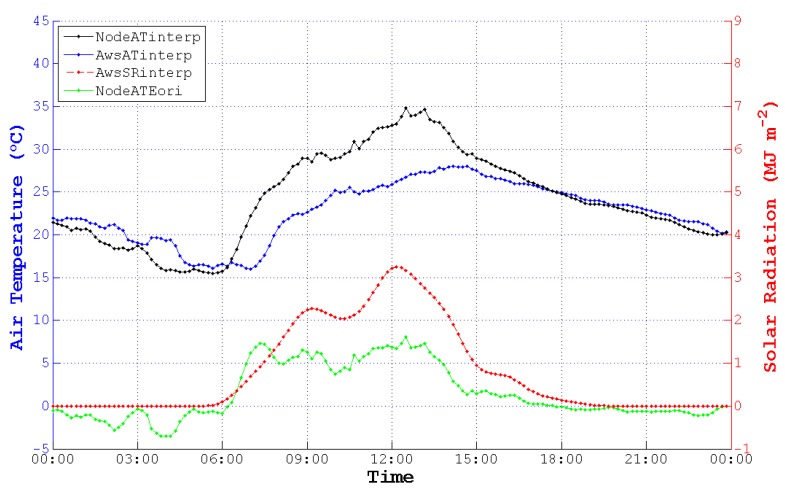
AT and SR after interpolating on 13 May 2014. NodeATinterp, AwsATinterp and AwsSRinterp are interpolated data corresponding to NodeAT, AwsAT and AwsSR. NodeATEori is the deviation between NodeATinterp and AwsATinterp.

Thus, cubic spline interpolation was used to unify the data resolution and to add sample points of NodeAT, AwsAT and AwsSR. As is depicted in Figure 8, NodeATinterp, AwsATinterp and AwsSRinterp were acquired with an identical time coordinate. Then, we obtained the original ATE between NodeATinterp and AwsATinterp (NodeATEori) to find the correlation between ATE and SR.

**Figure 9:**
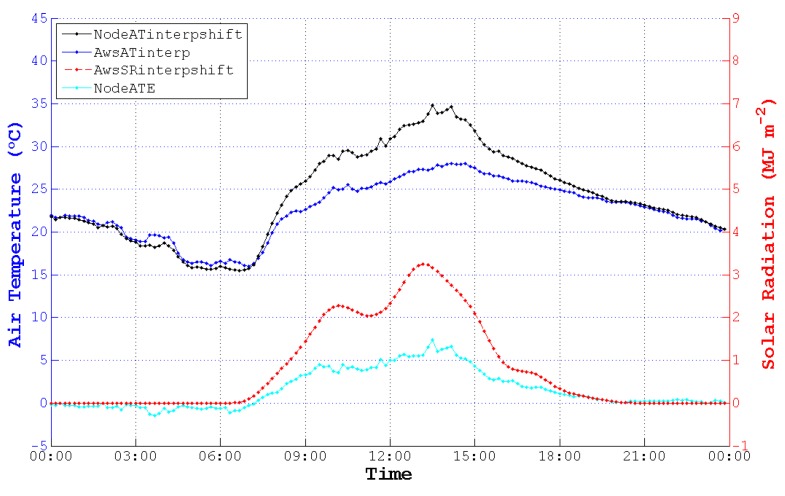
AT and SR after time shifting on 13 May 2014. NodeATinterpshift and AwsSRinterpshift are shifted data corresponding to NodeATinterp and AwsSRinterp. AwsATinterp is the interpolated data as before. NodeATE is the deviation between NodeATinterpshift and AwsATinterp.

However, as we can see in Figure 8, there is no obvious correlation between NodeATEori and AwsSRinterp. What is more, the values of NodeATEori are not close to zero, while AwsSRinterp are zero. However, theoretically speaking, the deviations between NodeAT and AwsAT should be zero when the value of SR is zero, for there is no solar radiation in the evening. For this reason, we tried to shift NodeATinterp and AwsSRinterp to the future by 60 min to approach AwsATinterp, as is plotted in Figure 9. It is easy to find that the pattern of NodeATinterpshift gets more correlated to AwsATinterp, and NodeATE is more relative to AwsSRinterpshift.

**Figure 10:**
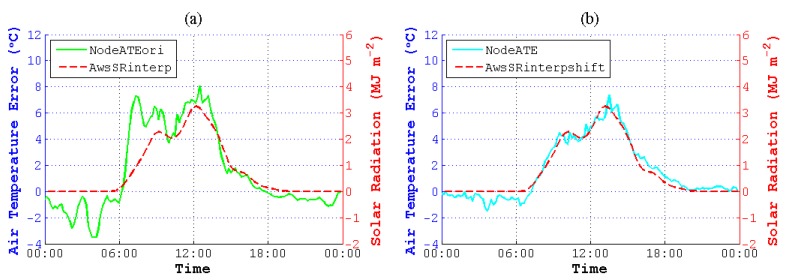
Contrast of AT error (ATE) and SR on 13 May 2014. (**a**) The enlarged ATE-SR pattern of Figure 8; (**b**) the enlarged ATE-SR pattern of Figure 9.

In order to make it more visual to observe the effect of shifting, we enlarged the ATE-SR patterns of Figure 8 and Figure 9 by narrowing the limit of the Y-axis, changed the Y-tick [46] of SR and put them into one figure. As is displayed in Figure 10, NodeATE, which is obtained after time shifting, changes along with the trend of SR visibly.

Then, the relationship of ATE and SR was acquired by calculating the average value of NodeATE in May corresponding to every value of SR (from 0.01 to 3.60, increasing by 0.01) using statistical analysis. The corresponding relation of ATE and SR is presented in Table 2.

Then, the ATE-SR function Err=A(r) was obtained according to the relationship. Therefore, it is feasible to work out CalcATE by using the value of SR as a parameter in Err=A(r).

To correct NodeAT, we followed three steps: (1) correcting the time coordinate of NodeAT by shifting them to the future by 60 min and getting NodeATshift; then (2) calculating CalcATE by using AwsSRshift as an input parameter in the ATE-SR function; and (3) obtaining the corrected data NodeATcorr by subtracting CalcATE from NodeATshift.

**Figure 11:**
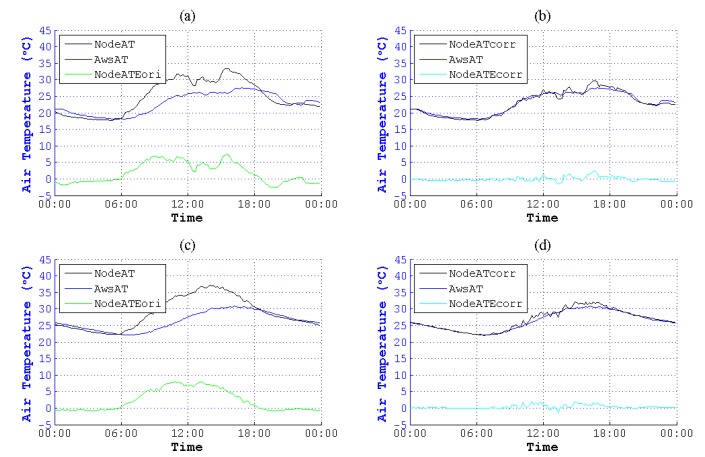
Contrast of NodeAT and NodeATcorr on 4 June 2014 and 9 June 2014. (**a**) Original data on 4 June 2014; (**b**) corrected data on 4 June 2014; (**c**) original data on 9 June 2014; (**d**) corrected data on 9 June 2014.

We used this method to correct NodeAT in June to December in different seasons. As is plotted in Figure 11 to Figure 17, NodeATcorr approximates AwsAT very well. Obviously, this method is useful to reduce the data error of NodeAT.

**Figure 12:**
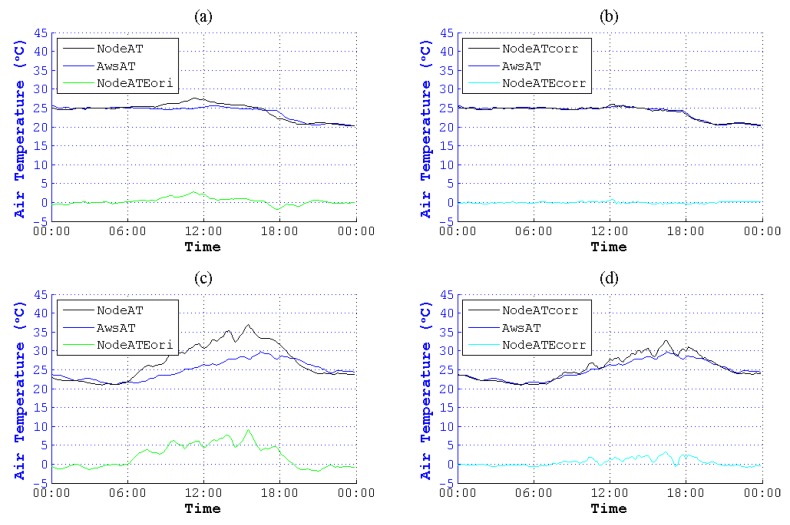
Contrast of NodeAT and NodeATcorr on 4 July 2014 and 7 July 2014. (**a**) Original data on 4 July 2014; (**b**) corrected data on 4 July 2014; (**c**) original data on 7 July 2014; (**d**) corrected data on 7 July 2014.

**Table 2:** ATE values corresponding to every possible value of SR in May.

**SR (MJ·m^−2^)**	0.01	0.02	0.03	0.04	0.05	0.06	0.07	0.08	0.09	0.10	0.11	0.12	0.13	0.14	0.15	0.16	0.17	0.18	0.19	0.20
**ATE (°C)**	0.01	0.01	0.12	0.18	0.31	0.23	0.39	0.34	0.40	0.37	0.37	0.62	0.42	0.54	0.53	0.72	0.35	0.59	0.79	0.67
**SR (MJ·m^−2^)**	0.21	0.22	0.23	0.24	0.25	0.26	0.27	0.28	0.29	0.30	0.31	0.32	0.33	0.34	0.35	0.36	0.37	0.38	0.39	0.40
**ATE (°C)**	0.67	0.65	0.74	0.86	0.73	0.92	0.84	0.87	1.16	0.99	0.98	0.72	0.97	0.81	0.92	1.15	1.25	1.10	1.26	1.18
**SR (MJ·m^−2^)**	0.41	0.42	0.43	0.44	0.45	0.46	0.47	0.48	0.49	0.50	0.51	0.52	0.53	0.54	0.55	0.56	0.57	0.58	0.59	0.60
**ATE (°C)**	1.13	1.13	1.13	1.61	1.27	1.28	1.55	1.54	1.31	1.51	1.62	1.55	1.41	1.72	1.65	1.51	1.56	1.72	1.31	1.64
**SR (MJ·m^−2^)**	0.61	0.62	0.63	0.64	0.65	0.66	0.67	0.68	0.69	0.70	0.71	0.72	0.73	0.74	0.75	0.76	0.77	0.78	0.79	0.80
**ATE (°C)**	1.63	1.63	2.21	1.58	1.72	1.98	1.93	1.88	1.85	1.66	1.74	1.77	1.74	2.13	2.05	2.56	2.02	2.29	2.25	2.14
**SR (MJ·m^−2^)**	0.81	0.82	0.83	0.84	0.85	0.86	0.87	0.88	0.89	0.90	0.91	0.92	0.93	0.94	0.95	0.96	0.97	0.98	0.99	1.00
**ATE (°C)**	1.86	2.17	2.08	2.48	2.13	2.40	2.30	2.16	2.31	2.53	2.26	2.28	2.33	2.10	2.79	2.02	2.29	2.46	2.36	2.54
**SR (MJ·m^−2^)**	1.01	1.02	1.03	1.04	1.05	1.06	1.07	1.08	1.09	1.10	1.11	1.12	1.13	1.14	1.15	1.16	1.17	1.18	1.19	1.20
**ATE (°C)**	2.73	2.15	2.44	2.31	2.82	2.56	2.26	2.26	2.33	2.90	2.83	2.88	2.67	2.92	2.92	2.62	2.74	2.81	2.83	3.05
**SR (MJ·m^−2^)**	1.21	1.22	1.23	1.24	1.25	1.26	1.27	1.28	1.29	1.30	1.31	1.32	1.33	1.34	1.35	1.36	1.37	1.38	1.39	1.40
**ATE (°C)**	3.45	2.72	3.03	2.91	2.82	3.38	2.87	2.95	2.81	2.94	2.75	2.89	2.88	3.38	2.98	3.49	3.68	3.36	3.40	3.33
**SR (MJ·m^−2^)**	1.41	1.42	1.43	1.44	1.45	1.46	1.47	1.48	1.49	1.50	1.51	1.52	1.53	1.54	1.55	1.56	1.57	1.58	1.59	1.60
**ATE (°C)**	3.20	3.26	3.02	2.98	3.32	3.38	3.27	3.64	3.26	3.58	3.64	3.64	2.99	3.32	3.33	3.71	3.11	3.72	3.34	3.34
**SR (MJ·m^−2^)**	1.61	1.62	1.63	1.64	1.65	1.66	1.67	1.68	1.69	1.70	1.71	1.72	1.73	1.74	1.75	1.76	1.77	1.78	1.79	1.80
**ATE (°C)**	3.42	4.02	3.45	4.15	3.77	4.17	3.18	4.11	3.46	3.72	3.28	2.96	2.96	3.78	4.04	4.04	3.78	3.90	3.55	3.81
**SR (MJ·m^−2^)**	1.81	1.82	1.83	1.84	1.85	1.86	1.87	1.88	1.89	1.90	1.91	1.92	1.93	1.94	1.95	1.96	1.97	1.98	1.99	2.00
**ATE (°C)**	3.60	3.60	3.60	4.37	4.02	3.66	4.27	3.98	3.55	4.00	4.12	4.12	3.58	3.06	4.02	3.62	4.16	3.47	3.85	4.55
**SR (MJ·m^−2^)**	2.01	2.02	2.03	2.04	2.05	2.06	2.07	2.08	2.09	2.10	2.11	2.12	2.13	2.14	2.15	2.16	2.17	2.18	2.19	2.20
**ATE (°C)**	4.16	4.12	3.45	4.29	4.19	4.22	4.11	4.24	4.49	3.43	4.80	4.44	3.97	3.87	4.20	4.83	4.26	4.64	4.73	4.47
**SR (MJ·m^−2^)**	2.21	2.22	2.23	2.24	2.25	2.26	2.27	2.28	2.29	2.30	2.31	2.32	2.33	2.34	2.35	2.36	2.37	2.38	2.39	2.40
**ATE (°C)**	4.56	4.23	4.65	3.97	3.61	4.84	4.10	4.21	4.77	5.05	5.00	3.60	4.72	4.99	3.87	5.08	4.83	4.04	5.08	4.82
**SR (MJ·m^−2^)**	2.41	2.42	2.43	2.44	2.45	2.46	2.47	2.48	2.49	2.50	2.51	2.52	2.53	2.54	2.55	2.56	2.57	2.58	2.59	2.60
**ATE (°C)**	4.40	5.14	3.97	4.69	4.62	3.82	4.42	4.91	4.65	5.30	4.98	5.34	5.53	4.62	5.47	5.13	5.07	5.18	5.26	4.34
**SR (MJ·m^−2^)**	2.61	2.62	2.63	2.64	2.65	2.66	2.67	2.68	2.69	2.70	2.71	2.72	2.73	2.74	2.75	2.76	2.77	2.78	2.79	2.80
**ATE (°C)**	5.48	4.75	4.88	5.61	5.79	5.16	4.81	4.81	4.78	5.14	5.70	5.15	5.13	5.59	5.72	5.31	5.08	5.07	4.84	5.36
**SR (MJ·m^−2^)**	2.81	2.82	2.83	2.84	2.85	2.86	2.87	2.88	2.89	2.90	2.91	2.92	2.93	2.94	2.95	2.96	2.97	2.98	2.99	3.00
**ATE (°C)**	5.40	5.17	5.56	4.90	6.48	5.75	4.98	4.32	5.17	5.07	5.06	5.16	6.06	5.70	6.00	4.61	6.05	5.72	6.04	5.59
**SR (MJ·m^−2^)**	3.01	3.02	3.03	3.04	3.05	3.06	3.07	3.08	3.09	3.10	3.11	3.12	3.13	3.14	3.15	3.16	3.17	3.18	3.19	3.20
**ATE (°C)**	6.06	5.17	5.26	6.22	5.52	5.61	5.41	6.11	5.01	5.67	5.47	6.61	5.89	5.39	6.49	6.21	6.60	6.05	5.09	7.38
**SR (MJ·m^−2^)**	3.21	3.22	3.23	3.24	3.25	3.26	3.27	3.28	3.29	3.30	3.31	3.32	3.33	3.34	3.35	3.36	3.37	3.38	3.39	3.40
**ATE (°C)**	5.64	5.90	5.82	6.70	5.60	5.54	5.54	5.91	5.49	6.29	4.84	5.37	6.26	4.72	5.93	5.43	5.43	5.60	6.56	5.99
**SR (MJ·m^−2^)**	3.41	3.42	3.43	3.44	3.45	3.46	3.47	3.48	3.49	3.50	3.51	3.52	3.53	3.54	3.55	3.56	3.57	3.58	3.59	3.60
**ATE (°C)**	5.76	5.66	6.36	6.50	6.32	6.55	5.48	5.48	6.26	6.26	4.96	4.96	4.96	6.36	5.23	5.78	5.78	5.18	6.32	////

**Figure 13:**
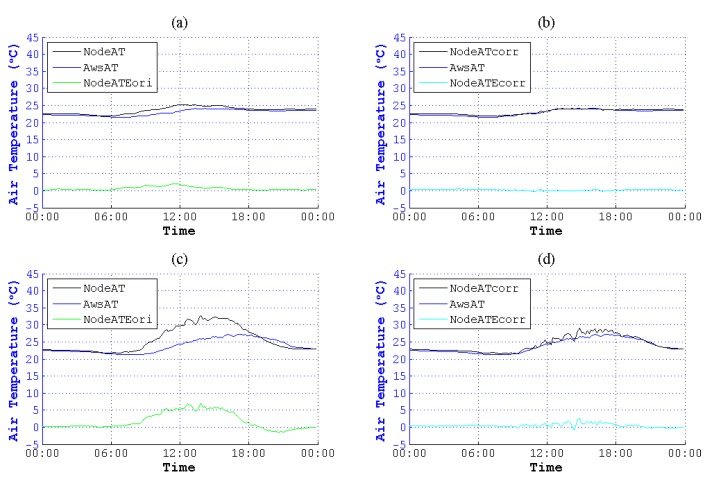
Contrast of NodeAT and NodeATcorr on 8 August 2014 and 10 August 2014. (**a**) Original data on 8 August 2014; (**b**) corrected data on 8 August 2014; (**c**) original data on 10 August 2014; (**d**) corrected data on 10 August 2014.

**Figure 14:**
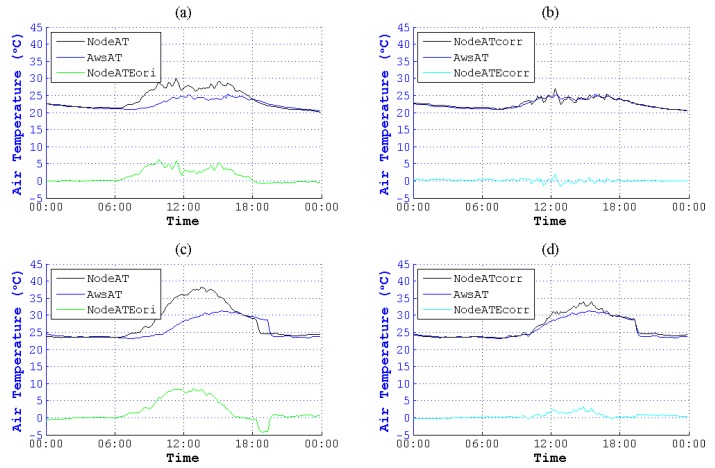
Contrast of NodeAT and NodeATcorr on 3 September 2014 and 28 September 2014. (**a**) Original data on 3 September 2014; (**b**) corrected data on 3 September 2014; (**c**) original data on 28 September 2014; (**d**) corrected data on 28 September 2014.

**Figure 15:**
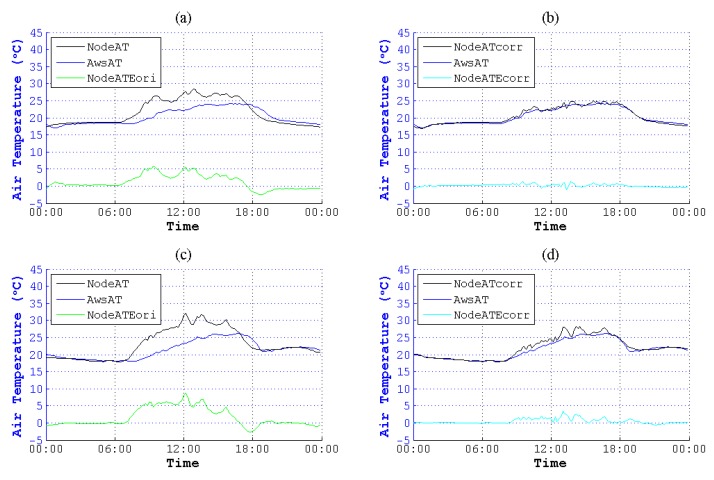
Contrast of NodeAT and NodeATcorr on 2 October 2014 and 19 October 2014. (**a**) Original data on 2 October 2014; (**b**) corrected data on 2 October 2014; (**c**) original data on 19 October 2014; (**d**) corrected data on 19 October 2014.

**Figure 16:**
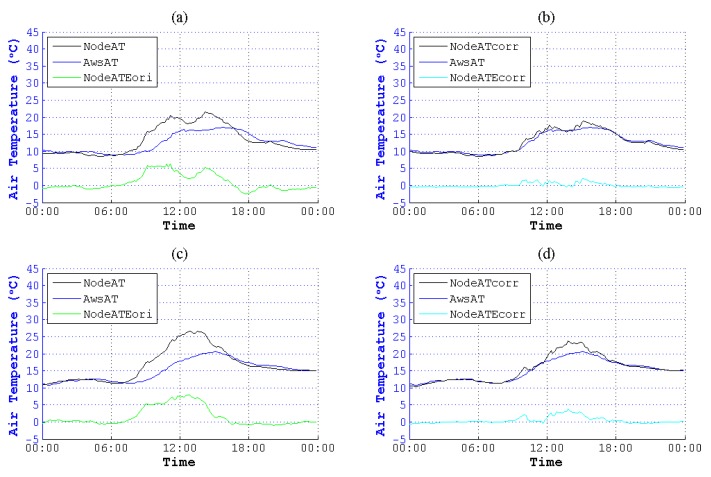
Contrast of NodeAT and NodeATcorr on 11 November 2014 and 22 November 2014. (**a**) Original data on 11 November 2014; (**b**) corrected data on 11 November 2014; (**c**) original data on 22 November 2014; (**d**) corrected data on 22 November 2014.

**Figure 17:**
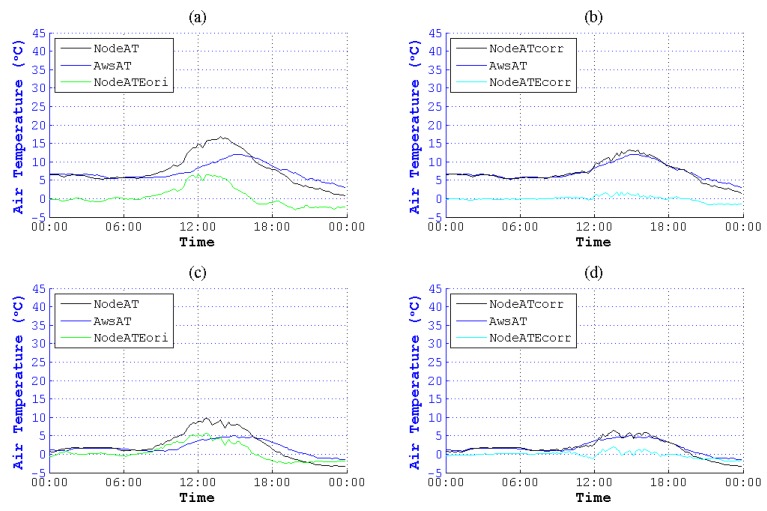
Contrast of NodeAT and NodeATcorr on 7 December 2014 and 21 December 2014. (**a**) Original data on 7 December 2014; (**b**) corrected data on 7 December 2014; (**c**) original data on 21 December 2014; (**d**) corrected data on 21 December 2014.

### 3.3. Performance Evaluations

We calculated several kinds of statistical characterizations to evaluate the performance of this correcting method. Maximal and mean error and the standard deviation of error were computed to estimate the correcting efficiency of AT error, and the correlation coefficient was given to show the degree of correlation between NodeAT and AwsAT in different correcting phases.

Error correcting efficiency can be obtained by following equation:

$$errorCorrectEfficiency=\frac{errorOriginal-errorCorrected}{errorOriginal}\times 100\%$$

To make the evaluation more objective, we used the absolute value of error to calculate the maximal error and mean error, and the applied error contains a negative value to calculate the standard deviation.

As we can see in Table 3 and Table 4, the values of maximal error and mean error are decreasing progressively along with the correcting course. Comparing these two statistical characterizations before shifting with these after shifting, we can find that the process of time shifting can reduce some error. This means that time correction is effective to reduce some of the error. More values of the error are cut down by the uppermost value correction. Finally, the error had been reduced largely by these two correcting process.

**Table 3:** Maximal absolute error in different correcting phases.

	Maximal Absolute Error			
Date	Original Data	Time Shifted	Value Corrected	Correcting Efficiency
4 June 2014	7.54	6.00	2.35	69%
9 June 2014	8.13	6.76	2.00	75%
4 July 2014	2.83	2.33	0.70	75%
7 July 2014	9.04	7.14	3.32	63%
8 August 2014	2.07	1.37	0.54	74%
10 August 2014	7.05	6.40	2.53	64%
3 September 2014	6.21	5.01	2.02	67%
28 September 2014	8.45	7.49	3.16	63%
2 October 2014	5.92	4.62	1.42	76%
19 October 2014	8.64	7.07	3.52	59%
11 November 2014	6.35	4.72	2.05	68%
22 November 2014	7.96	6.76	3.77	53%
7 December 2014	6.56	5.26	1.89	71%
21 December 2014	5.76	5.16	2.09	64%
**Average**	**6.61**	**5.44**	**2.24**	**66%**

**Table 4:** Mean absolute error in different correcting phases.

	Mean Absolute Error			
Date	Original Data	Time Shifted	Value Corrected	Correcting Efficiency
4 June 2014	2.78	2.12	0.51	82%
9 June 2014	2.81	2.35	0.42	85%
4 July 2014	0.71	0.62	0.19	73%
7 July 2014	2.86	2.42	0.82	72%
8 August 2014	0.67	0.62	0.25	62%
10 August 2014	2.08	1.83	0.54	74%
3 September 2014	1.62	1.50	0.31	81%
28 September 2014	2.41	1.99	0.64	73%
2 October 2014	1.85	1.37	0.37	80%
19 October 2014	2.16	1.72	0.59	73%
11 November 2014	1.83	1.28	0.49	73%
22 November 2014	1.97	1.55	0.67	66%
7 December 2014	1.95	1.33	0.53	73%
21 December 2014	1.80	1.37	0.60	67%
**Average**	**1.96**	**1.58**	**0.50**	**74%**

Table 5 presents the standard deviation of error on every whole day. The values of the standard deviation are also decreasing progressively along with every step of correction.

**Table 5:** Standard deviation of the error in different correcting phases.

	Standard Deviation of Error			
Date	Original Data	Time Shifted	Value Corrected	Correcting Efficiency
4 June 2014	3.05	2.30	0.66	78%
9 June 2014	3.23	2.52	0.55	83%
4 July 2014	0.89	0.66	0.23	74%
7 July 2014	3.07	2.54	0.99	68%
8 August 2014	0.50	0.32	0.18	63%
10 August 2014	2.45	1.99	0.52	79%
3 September 2014	1.93	1.52	0.42	78%
28 September 2014	3.21	2.46	0.74	77%
2 October 2014	2.08	1.50	0.42	80%
19 October 2014	2.77	2.08	0.75	73%
11 November 2014	2.38	1.66	0.64	73%
22 November 2014	2.79	2.21	1.01	64%
7 December 2014	2.66	1.91	0.76	71%
21 December 2014	2.30	1.82	0.81	65%
**Average**	**2.38**	**1.82**	**0.62**	**74%**

**Table 6:** Correlation coefficient between NodeAT and AweAT in three different correcting phases.

	Correlation Coefficient		
Date	Original Data	Time Shifted	Value Corrected
4 June 2014	0.8221	0.9496	0.9870
9 June 2014	0.7518	0.9037	0.9870
4 July 2014	0.9187	0.9483	0.9910
7 July 2014	0.8227	0.9487	0.9785
8 August 2014	0.8729	0.9605	0.9784
10 August 2014	0.7828	0.9252	0.9794
3 September 2014	0.8209	0.9666	0.9531
28 September 2014	0.8033	0.9506	0.9859
2 October 2014	0.8575	0.9779	0.9879
19 October 2014	0.7837	0.9338	0.9777
11 November 2014	0.8390	0.9666	0.9871
22 November 2014	0.8303	0.9491	0.9791
7 December 2014	0.8393	0.9709	0.9860
21 December 2014	0.8663	0.9700	0.9696
**Average**	**0.8294**	**0.9515**	**0.9806**

In Table 6, we present the Pearson product-moment correlation coefficient between NodeAT and AweAT in three different phases. In statistics, the Pearson product-moment correlation coefficient (sometimes referred to as the PPMCC, or PCC, or Pearson’s r) is a measure of the linear correlation (dependence) between two variables *X* and *Y*, giving a value between +1 and −1 inclusive, where 1 is the total positive correlation, 0 is no correlation and −1 is total negative correlation [48]. It is widely used in the sciences as a measure of the degree of linear dependence between two variables and can be used to measure the correlation between NodeAT and AwsAT. As we can see in the column “Original Data” in Table 6, the correlation coefficients between uncorrected NodeAT and standard AwsAT are not high. This means a low correlation degree between unprocessed data and standard data. However, the coefficient was improved after time shifting, and further advanced after the process of value correcting. Eventually, there is a higher correlation coefficient between corrected NodeAT and standard AwsAT.

## 4. Conclusions

In this paper, an effective error correcting method for NodeAT is presented. According to the results, more than 60% of the error of NodeAT can be corrected by using this approach, and it can be applied to the real-time AT monitoring system in a practical scenario.

This study has confirmed that SR plays an extremely vital role in the correcting scheme of NodeAT. However, the ATE-SR function, which is based on discrete data, potentially can be perfected. What is more, this method has to rely on the data of SR sensed by a pyranometer. The cost of SR sensing is still high. In order to reduce the expense of our project further, the data of the voltage of the solar cell panel equipped in the sensing node will be considered to replace the data of SR in the future.

## Appendix

### A. Radiation and Solar Radiation

#### A.1. Radiation

Solar radiation arriving on a surface is variably termed irradiation, insolation, radiation, irradiance, radiance, intensity, radiant flux, radiant flux density, *etc*. [44]. Irradiation and insolation are used interchangeably in this paper, both referring to the quantity of solar energy arriving at a surface during a given period of time. Literally, irradiation simply refers to radiation arriving at a surface, whether or not the origin of radiation is the Sun. Irradiance will indicate the rate of solar energy arriving at a surface per unit time and per unit area. Irradiance is the same as radiant flux density or flux. Since irradiance will mean the rate of incident energy, its units will be W·m${}^{-2}$, and the units of irradiation will be kJ·m${}^{-2}$·h${}^{-1}$ or MJ·m${}^{-2}$·day${}^{-1}$. Radiation will be employed in a generic sense, and its meaning should be treated as irradiation in this paper. Intensity means irradiance from a particular direction and contained within a unit solid angle. Intensity is expressed in W·m${}^{-2}$·sr${}^{-1}$ on an area normal to the direction of radiation. It is pertinent to point out that the term “intensity” is often loosely employed. For example, in meteorology, intensity is used for radiative flux, as well as for the quantity of radiation arriving from all over the sky dome [44].

#### A.2. Solar Radiation

The Sun is the star closest to Earth, and its radiant energy is practically the only source of energy that influences atmospheric motions and our climate [44]. Due to solar radiation emanating from the Sun being attenuated before reaching the ground, the maximum radiation on the Earth is received under cloudless and clear sky [44].

When solar radiation enters the Earth’s atmosphere, a part of the incident energy is removed by scattering and a part by absorption. The scattered radiation is called diffuse radiation. A portion of this diffuse radiation goes back to space, and a portion reaches the ground. The radiation arriving on the ground directly in line of the solar disk is called direct or beam radiation [44]. The quantity of total direct and diffuse radiation arriving at the Earth’s surface is important to the temperature variation [49] on the ground. However, in order to quantify SR in a horizontal surface, global solar irradiance has been presented. Global irradiance is the sum of the beam plus diffuse irradiance on a horizontal surface and can be measured by radiometers with hemispherical fields of view, called pyranometers [44]. Additionally, global solar irradiation is the integral of solar irradiance in the period of the given time interval.

### B. Interpolation and Spline Functions

#### B.1. Interpolation

Suppose that the function y=f(x) is known at the N+1 points $\left(x_{0},y_{0}\right)$, ..., $\left(x_{N},y_{N}\right)$, where the values $x_{k}$ are spread out over the interval [a,b] and satisfy:

$$a\leq x_{0}<x_{1}<\cdots <x_{N}\leq b\;\text{and}\;y_{k}=f\left(x_{k}\right)$$

A polynomial P(x) of degree *N* will be constructed that passes through these N+1 points. In the construction, only the numerical values $x_{k}$ and $y_{k}$ are needed. Hence, the higher-order derivatives are not necessary. The polynomial P(x) can be used to approximate f(x) over the entire interval [a,b] [50].

Situations in statistical and scientific analysis arise where the function y=f(x) is available only at N+1 tabulated points $\left(x_{k},y_{k}\right)$, and a method is needed to approximate f(x) at non-tabulated abscissas. When $x_{0}<x<x_{N}$, the approximation P(x) is called an interpolated value. If either $x<x_{0}$ or $x_{N}<x$, then P(x) is called an extrapolated value [50].

#### B.2. Spline Function

A spline function is a function that consists of polynomial pieces joined together with certain smoothness conditions [51].

In most situations, polynomial interpolation for a set of N+1 points ${\left\{\left(x_{k},y_{k}\right)\right\}}_{k=0}^{N}$ is frequently unsatisfactory from a practical point of view, and other functions need to be considered [50,51].

Another method is to piece together the graphs of lower-degree polynomials $S_{k}\left(x\right)$ and interpolate between the successive nodes $\left(x_{k},y_{k}\right)$ and $\left(x_{k+1},y_{k+1}\right)$. The two adjacent portions of the curve $y=S_{k}\left(x\right)$ and $y=S_{k+1}\left(x\right)$, which lie above $\left[x_{k},x_{k+1}\right]$ and $\left[x_{k+1},x_{k+2}\right]$, respectively, pass through the common knot $\left(x_{k+1},y_{k+1}\right)$, and the set of functions $\left\{S_{k}\left(x\right)\right\}$ forms a piecewise polynomial curve, which is denoted by S(x) [50].

According to the degree of piecewise polynomial, spline function can be classified as first-degree spline (whose pieces are linear polynomials joined together to achieve continuity) [51], quadratic spline (which is a continuously differentiable piecewise quadratic function, where quadratic includes all linear combinations of the basic monomials $x\mapsto 1,x,x^{2}$) [51,52], cubic spline [51,53] and higher-degree spline [51]. Compared with splines of other degrees, the cubic spline function has two continuous derivatives everywhere. At each knot, three continuity conditions are imposed. Since *S*, $S^{’}$ and $S^{’’}$ are continuous, the graph of the function will appear smooth to the eye. Discontinuities, of course, may occur in the third derivative, but cannot be easily detected visually, which is one reason for choosing degree three. Experience has shown, moreover, that using splines of a degree greater than three seldom yields any advantage. For technical reasons, odd-degree splines behave better than even-degree splines (when interpolating at the knots) [51].
